# Effects of dynamic quarantine and nonlinear infection rate in a model for computer worms propagation

**DOI:** 10.1063/1.4912581

**Published:** 2015-03-10

**Authors:** Carla M.A. Pinto

**Affiliations:** School of Engineering, Polytechnic of Porto, and Centre for Mathematics, University of Porto, Rua Dr António Bernardino de Almeida, 431, 4200-072 Porto, Portugal cap@isep.ipp.pt; King Saud University, Riyadh, Saudi Arabia and University of Peloponnese, Tripolis, Greece; TEI of Sterea Hellas, Psahna, Greece

## Abstract

We propose a new model for computer worms propagation, using dynamic quarantine and a nonlinear infection rate. The dynamic quarantine is based in epidemic disease control methods and in the principle ‘assume guilty before proven inocent’. This means that the host is blocked whenever its behavior looks suspicious. After a short time, the quarantined computer is released. The nonlinear infection rate is used to capture the dynamics of overcrowded infectious networks and high viral loads. We simulate numerically the model for distinct values of the quarantine times. We observe that increasing the quarantine time decreases the number of infectious hosts in the network.

### INTRODUCTION

Computer worms propagation has been a major research topic for a considerably large number of researchers, in the last few decades. The extraordinary increase in the number of internet users, with the consequential increase in the number of internet communications, provided a good environment for worms to spread. The propagation of worms is highly damaging, translating in losses of millions of dollars and disrupted productivity [7].

The characteristics of computer worms transmission, in particular their resemblence with infectious diseases propagation, suggested the application of mathematical models for epidemics, to investigate the spreading of worms. Throughout the years, several compartmental models have been applied in this study, namely SI (Susceptible-Infectious), SIR (Susceptible-Infectious-Recovered), SEIR (Susceptible-Exposed-Infectious-Recovered), SIQV (Susceptible-Infectious-Quarantine-Vaccinated), SLB (Susceptible-Latent-Breaking), amongst others [4, 8, 11, 10].

The majority of mathematical models for worm propagation, considers constant quarantine [13, 8]. Nevertheless, this type of quarantine is innefficient, due to the high values of the rate at which new hosts entering the network are patched. Dynamic quarantine methods, based on the principle 'assume guilty before proven innocent' have been proposed to mitigate this problem [14, 9, 12]. This dynamic quarantine method diminishes the negative effect of false alarms, produced by worm anomaly detection systems. A host is quarantined whenever he has a suspicious behavior, and after some time he is released from quarantine. Once a host is quarantined, security assistants should inspect it as soon as possible. False quarantined hosts won't be blocked for a long period, since quarantine is released after some time. This dynamic quarantine method can be built on any worm anomaly detection systems. Other quarantine measures, such as pulse quarantine, have been proposed recently in the literature, due to optimistic results from epidemic models using pulse vaccination [11]. Pulse vaccination allows systems to stabilize at disease-free equilibrium faster than constant vaccination. Pulse vaccination will be the focus of future work.

The incidence rate is extremely important in the modeling of disease dynamics. Usually this incidence rate is a function of the numbers of susceptible and infectious individuals. Nevertheless, these incidence rates are ineffective in the cases of overcrowded infectives and high viral loads. Moreover, the topology of the underlaying network may also affect the worm's spread, suggesting nonlinear infection rates [3, 5, 2]. In this work, we consider nonlinear incidence rates of the form $\beta \frac{IS}{1+I}$ [1], where $\beta I$ measures the infection force of the disease and $1/(1+I)$ models the inhibition of susceptible nodes due to rising viral prevalences.

Bearing these ideas in mind, the paper is structured as follows. In Section 'THE MODEL', we describe the model for worm propagation with pulse quarantine and nonlinear incidence rates. In Section 'NUMERICAL SIMULATIONS', we present numerical simulations of the model for distinct values of the quarantine time. Finally, in Section 'CON-

*Proceedings of the International Conference on Numerical Analysis and Applied Mathematics 2014 (ICNAAM-2014)*
AIP Conf. Proc. 1648, 350003-1–350003-4; doi: 10.1063/1.4912581
© 2015 AIP Publishing LLC 978-0-7354-1287-3/$30.00

**TABLE 1.** Parameter values used in the simulations of model (1).

| Parameter | Value |
|---|---|
| $p$ | 0.2 |
| $\phi$ | 0.5 |
| $\beta$ | 0.1 |
| $\mu$ | 0.00001 |
| $\omega$ | 0.00000006 |
| $\lambda_1$ | 0.2 |
| $\lambda_2$ | 0.00002315 |
| $T$ | 10 |
| $\gamma$ | 0.004 |

CLUSIONS', we state the main conclusions of this work and sketch future resaerch directions.

### THE MODEL

The computers are denoted by nodes and can be at one of four possible states: susceptible (S), infectious (I), recovered (R), and quarantined (Q). The transitions between states are modelled by the following system of ordinary differential equations:

$$\begin{array}{rcl} \frac{dS}{dt} &=& p\mu - f(I,S) - (\mu+\omega+q_2)S \\ \frac{dI}{dt} &=& f(I,S) - (q_1+\mu+\gamma)I \\ \frac{dR}{dt} &=& (1-p)\mu + \gamma I + \phi Q + \omega S - \mu R \\ \frac{dQ}{dt} &=& q_2 S + q_1 I - (\phi+\mu)Q \end{array} \quad (1)$$

where $f(I,S) = \frac{0.01I}{1+I}S$.
The susceptible computers join the network at a rate $\mu$, $1-p$ of which is patched and move to the recovered state, $R$. All computers 'die' at a rate $\mu$. The total number of hosts in this network is unchanged, since 'death' and 'birth' rates are the same.

Susceptible hosts, $S$, with security vulnerabilities, are infected by worms at a rate $f(I,S)$, and move to the infectious class, $I$, or are directly patched, at a rate $\omega$, and move to the recovered class, $R$. Infectious hosts may be manually patched at a rate $\gamma$ and move to class $R$. Both susceptible, $S$, and infectious, $I$, computers can be detected by the misuse detection system and then constantly quarantined at rates $q_2$ and $q_1$, respectively. These rates are given by:

$$q_1 = \frac{\lambda_1 T}{1+(\lambda_1+\psi)T}, \qquad q_2 = \frac{\lambda_2 T}{1+\lambda_2 T} \quad (2)$$

where parameters $\lambda_1$ and $\lambda_2$ describe, respectively, the quarantine probability of infected hosts and susceptible hosts, which are related to the intrusion detection system. We consider $\lambda_1 > \lambda_2$, since the effect of false positives concerning susceptible hosts has to be reduced. The computers at the quarantined state, $Q$, are vaccinated against worms, by repairing and then patching, at rate $\phi$.

The dynamic quarantine strategy applied here has two advantages. The first one deals with false positives. A false positive quarantined healthy host will be quarantined only for a short time. The second advantage is that higher false alarm rates are more tolerable than with constant quarantine, thus infected hosts may be detected earlier.

### NUMERICAL SIMULATIONS

In this section we simulate the model (1) for distinct values of the quarantine time $T$, and for distinct nonlinear incidence rates. The initial condition is $S(0) = 999990$, $I(0) = 10$, $R(0) = 0$, and $Q(0) = 0$. The parameter values are given in Table 1.

The value of $\lambda_1 = 0.2/sec$, for the quarantine rate of infectious hosts, means that, on average, an infectious host can propagate for more or less $5sec$ before it is detected and quarantined. The quarantine rate of susceptible computers is set to $\lambda_2 = 0.00002315/sec$, indicating that the worm anomaly detection program will give on average twice false alarms for a healthy host per day.

In Figure 1, we depict the dynamics of the variables of model (1) for $T = 10$.

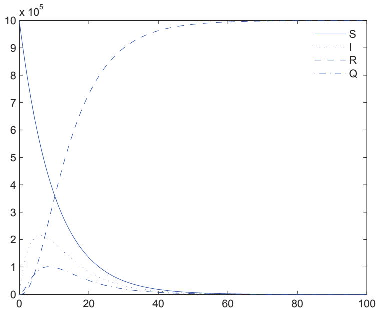

**FIGURE 1.** Dynamics of the variables of model (1) for $T = 10$. The model approaches a stable worm-free equilibrium.

Figures 2-3 depict the dynamics of the variables of model (1) for distinct values of $T$. We observe that as $T$ increases the system approaches faster the worm-free equilibrium. We need to find a commitment between the 'optimal' quarantine time and the least amount of time a host should be quarantined in real systems, to reduce the effects of false positives.

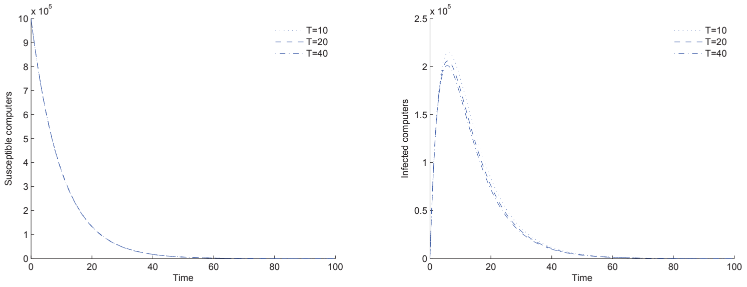

**FIGURE 2.** Dynamics of the variables of model (1) for $T = 10,\ 20,\ 40$. The model approaches a stable worm-free equilibrium. Note that as $T$ increases the system approaches faster the worm-free equilibrium.

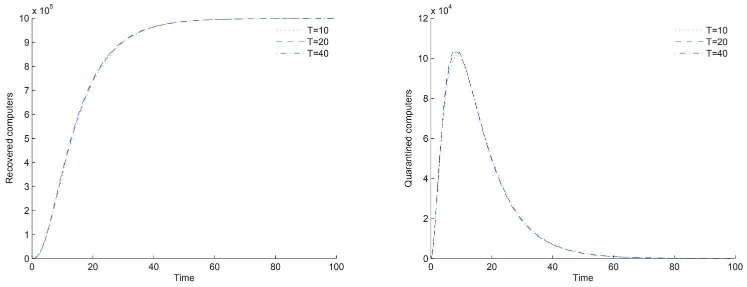

**FIGURE 3.** Dynamics of the variables of model (1) for $T = 10,\ 20,\ 40$. The model approaches a stable worm-free equilibrium. Note that as $T$ increases the system approaches faster the worm-free equilibrium.

## CONCLUSIONS

We study a model for computer worm propagation that includes dynamic quarantine and nonlinear incidence rate. We simulate numerically the model for distinct values of the quarantine time, $T$. We observe that as $T$ increases, the model approaches asymptotically faster the worm-free equilibrium. Future work will focus on the analysis of the stability of equilibria and the use of other nonlinear incidence rates.

### ACKNOWLEDGMENTS

The authors wish to thank Fundação Gulbenkian, through *Prémio Gulbenkian de Apoio à Investigação 2003*, Polytechnic of Porto, through the *PAPRE*, and the European Regional Development Fund through the program COMPETE and the Portuguese Government through the FCT - Fundação para a Ciência e a Tecnologia, under the project PEst-C/MAT/UI0144/2013 for financial support.
